# Augmented Reality in Neurosurgery, State of Art and Future Projections. A Systematic Review

**DOI:** 10.3389/fsurg.2022.864792

**Published:** 2022-03-11

**Authors:** Delia Cannizzaro, Ismail Zaed, Adrian Safa, Alice J. M. Jelmoni, Antonio Composto, Andrea Bisoglio, Kyra Schmeizer, Ana C. Becker, Andrea Pizzi, Andrea Cardia, Franco Servadei

**Affiliations:** ^1^Department of Neurosurgery, IRCCS Humanitas Research Hospital, Rozzano, Italy; ^2^Department of Biomedical Sciences, Humanitas University, Pieve Emanuele, Italy

**Keywords:** augmented reality, neurosurgery, education, training, cranial surgery, spine surgery

## Abstract

**Background:**

The use of augmented reality (AR) is growing in medical education, in particular, in radiology and surgery. AR has the potential to become a strategic component of neurosurgical training courses. In fact, over the years, there has been a progressive increase in the application of AR in the various fields of neurosurgery. In this study, the authors aim to define the diffusion of these augmented reality systems in recent years. This study describes future trends in augmented reality for neurosurgeons.

**Methods:**

A systematic review of the literature was conducted to identify research published from December 1st, 2011 to November 30th, 2021. Electronic databases (PubMed, PubMed Central, and Scopus) were screened. The methodological quality of studies and extracted data were assessed for “augmented reality” and “neurosurgery”. The data analysis focused on the geographical distribution, temporal evolution, and topic of augmented reality in neurosurgery.

**Results:**

A total of 198 studies have been included. The number of augmented reality applications in the neurosurgical field has increased during the last 10 years. The main topics on which it is mostly applied are spine surgery, neuronavigation, and education. The geographical distribution shows extensive use of augmented reality in the USA, Germany, China, and Canada. North America is the continent that uses augmented reality the most in the training and education of medical students, residents, and surgeons, besides giving the greatest research contribution in spine surgery, brain oncology, and surgical planning. AR is also extensively used in Asia for intraoperative navigation. Nevertheless, augmented reality is still far from reaching Africa and other countries with limited facilities, as no publications could be retrieved from our search.

**Conclusions:**

The use of AR is significantly increased in the last 10 years. Nowadays it is mainly used in spine surgery and for neurosurgical education, especially in North America, Europe and China. A continuous growth, also in other aspects of the specialty, is expected in the next future.

## Introduction

Augmented reality (AR) is a general terminology used to define a set of different technologies, all aiming to project virtual content into the real environment (1). In the past years, AR allowed for expanding the limits posed by two-dimensional imaging technologies, providing an unprecedented user experience in widespread fields, ranging from education, simulation, and medical specialties such as surgery and radiology (2). Concerning the medical field, AR has been widely used in different specialties, such as anesthesia, orthopedic surgery, neurosurgery, ophthalmology, urology, general surgery, and oral and maxillofacial surgery (3). Neurosurgery has always been at the forefront of this technology from the beginning, and still gives the greatest contribution to the literature (4). Conventional navigation and imaging technologies have tremendously advanced the field of neurosurgery in the past decades, providing crucial two-dimensional (2D) images, that have educated and guided neurosurgeons all over the world. However, when the surgeon must meet the three-dimensional (3D) extension of matter, these technologies may cause a cumbersome surgical workflow. In AR, computer-generated information is superimposed onto the real environment (the surgical field) to give a 3D semi-immersive experience, and a more integrated vision of the patient's status. The injection of multimodal preoperative and/or intraoperative images into the AR environment (such as MRI, CT, tractography, angiography, or ultrasound) enriches the surgeon's ability to simultaneously process data of different categories, nonetheless of crucial importance. Furthermore, this interactive surgical manipulation and anatomy visualization, integrated with haptic feedback, can significantly strengthen the resident's procedural memory and confidence during the procedure, also reducing the operation time (1, 2). These motivations clearly explain why AR has such great potential to become an essential part of neurosurgical training courses, starting from the earliest stages of a medical student's education to the training of an experienced neurosurgeon. Particularly in neurosurgery, where surgical corridors are often narrow and the margins of error are extremely low, AR has participated in revolutionary applications and brought major advances in all its sub specialties, ranging from the reduction in radiation exposure (5) and revision surgeries (6) to the safety and precision of neuro-oncologic resections (7).

With this systematic review, the authors aim to define the diffusion of AR in the world, highlighting some of the most critical challenges that should be addressed to introduce AR in routine clinical practice (8, 9). The analysis will be based on three layers: we will describe the geographical distribution of AR, the temporal evolution of the related publications in the past 10 years, and finally, we will analyze the relative trends in terms of research content, clinical applications, and education, which can provide crucial cues to predict the future of augmented reality.

## Materials and Methods

### Search Strategy

A systematic review has been conducted to achieve the aim of the study. A systemic broad search was done on PubMed using the search terms “augmented reality” and “neurosurgery” for the last 10 years, from December 1st, 2011 to December 31st, 2021. A broad search of 2 different medical databases (Pubmed and Scopus) has been conducted in order to identify articles that describe the use of AR in neurosurgery. In order to retrieve all the possible articles of interest, several keywords have been included: “augmented reality,” “neurosurgery.” These were combined with Boolean characters “AND” as well as “OR.” References from included articles were manually checked for proper additional studies.

### Study Selection

Several inclusion and exclusion criteria have been adopted. The authors only included studies published between December 1st, 2011 and December 31st, 2021. The studies included should also contain a recipient of the proposed augmented reality in neurosurgery. All the studies that did not examine any form of AR that reported technique and outcome were excluded. The authors also excluded all studies not written in English. All the reviews and meta-analyses have been widely searched for other possible inclusions. A qualitative analysis of the articles was performed by three authors (A.C., A.C.B, A.J.M J.). Any uncertainty in study selection was resolved by consensus among all authors.

### Data Extraction and Quality Assessment

For all included papers, three reviewers (AP) extracted and categorized data into structured tables. Extracted data included bibliographic information, type of paper, stated methodology, description of topic of application, and any formal research methods used. Data from the articles that were selected for screening was collected and applied to a database that included author, title, year of publication, country, and topic. The included articles were organized into categories based on country of publication, year of publication, and their topic. Once the articles were organized, a correlation between the topic and year of publication along with the topic and country of publication was made.

## Results

**Figure 1:**
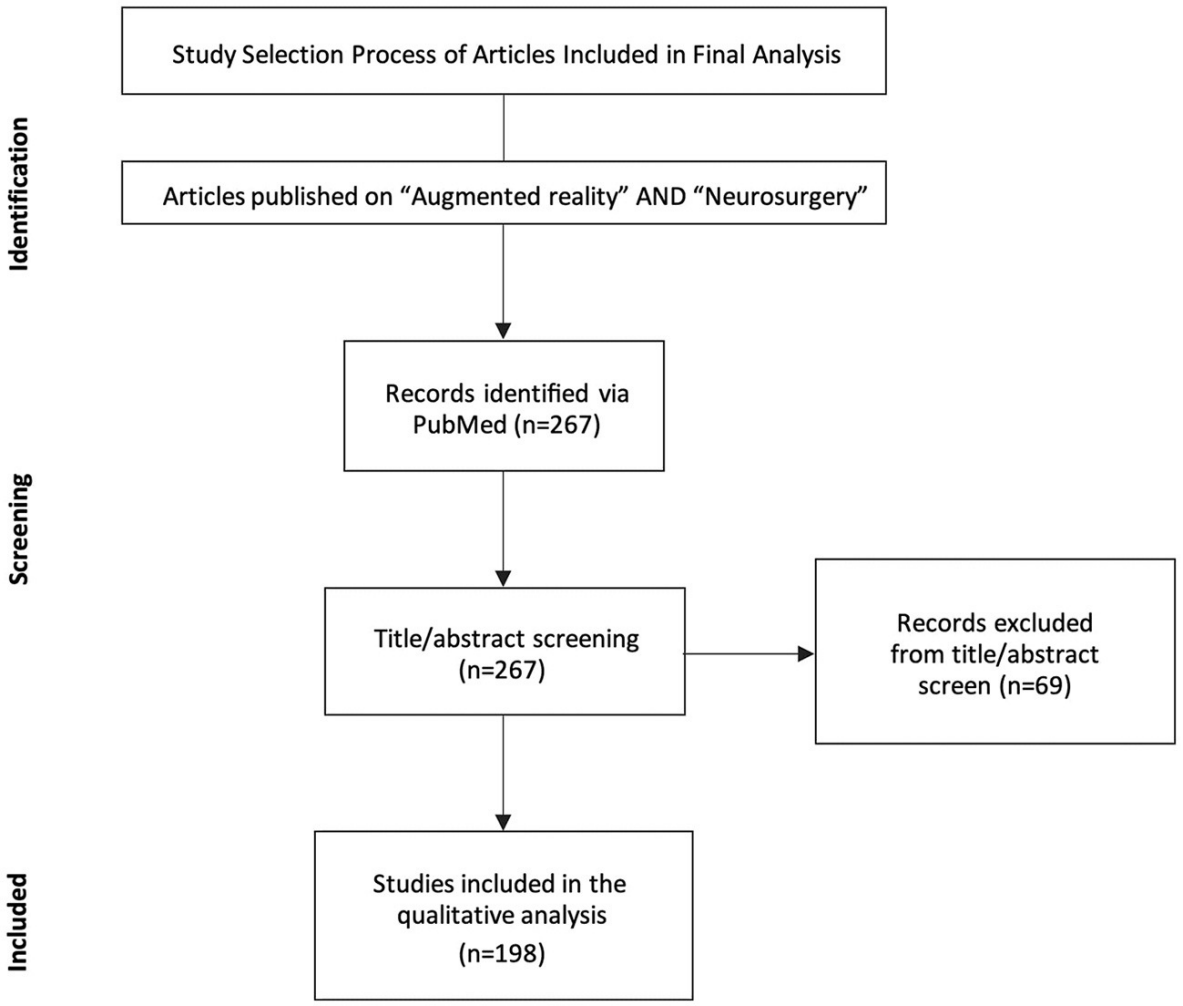
Prisma flow diagram of the literature search.

From 1 December 2011 to 30 November 2021, a total of 267 reports were identified by two authors (A.C., A.C.B.) using the above-mentioned methodology. After title screening, 14 articles were excluded. Of the 253 remaining, 55 articles were excluded after abstract screening, and 198 were included in the final analysis (Figure 1).

### Augmented Reality From 2011 to 2021

**Figure 2:**
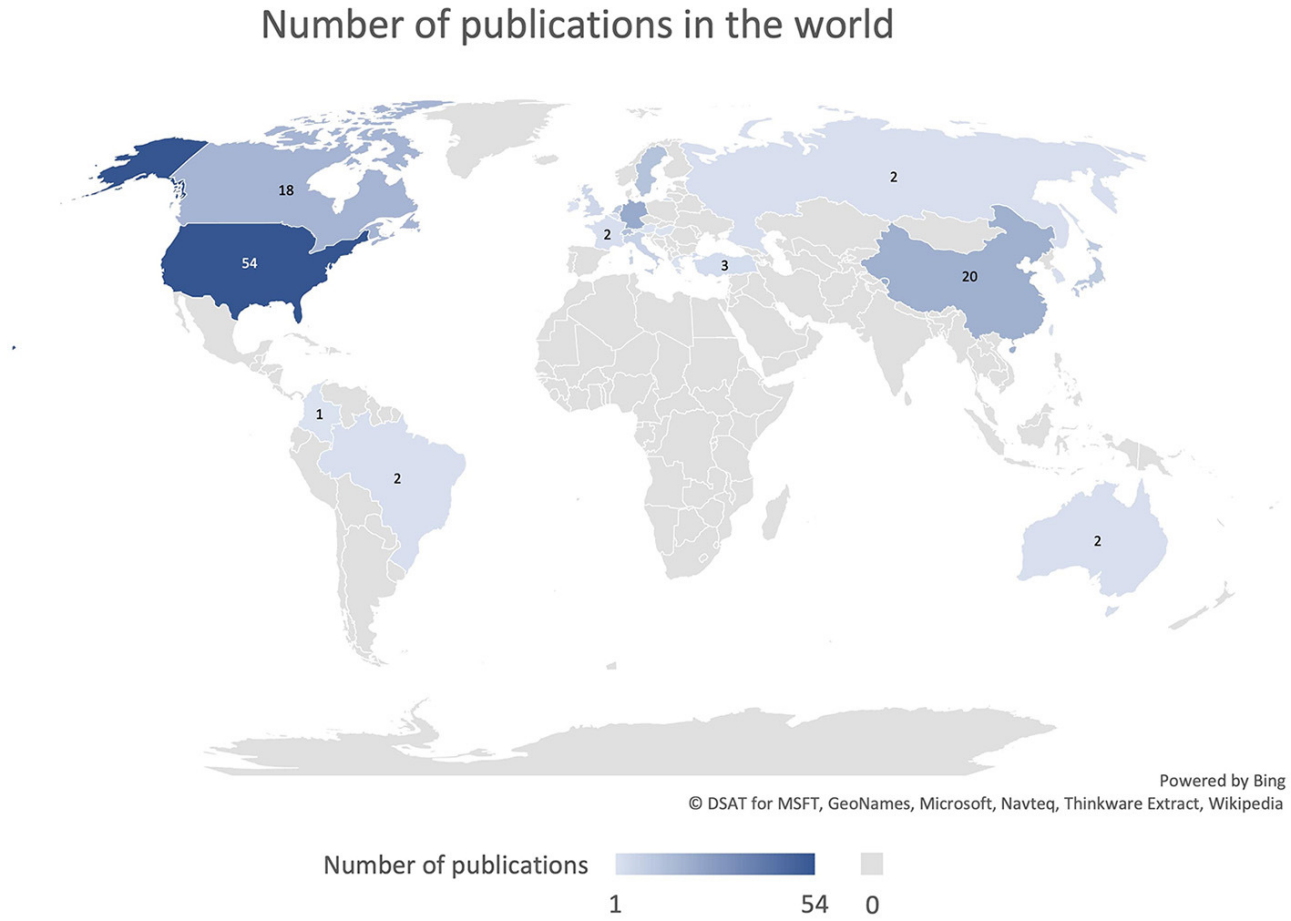
Diagram representing the number of publications per year.

The last 10 years have been characterized by a significant increase in the number of publications. The summary of the literature search has been shown in Figure 2 and reported in Table 1. Only 1 articles was published in 2012 about the use of AR in neurosurgery. As well shown in the table, in the first years, up to 2015 there has been a small interest on the topic, consisting in total publication of 24 articles. From 2016, there has been a consistent growing trend on the topic, which has gone from the 13 articles of 2016 up to the 34 studies in 2020, that has further exploded in 2021, resulting in 71 publications.

**Table 1:** Number of publications that could be retrieved from the search as grouped by the year of publication.

**Year**	**Number of publications**
2012	1
2013	6
2014	9
2015	8
2016	13
2017	19
2018	13
2019	24
2020	34
2021	71
Grand total	198

### Augmented Reality in Different Countries

**Figure 3:**
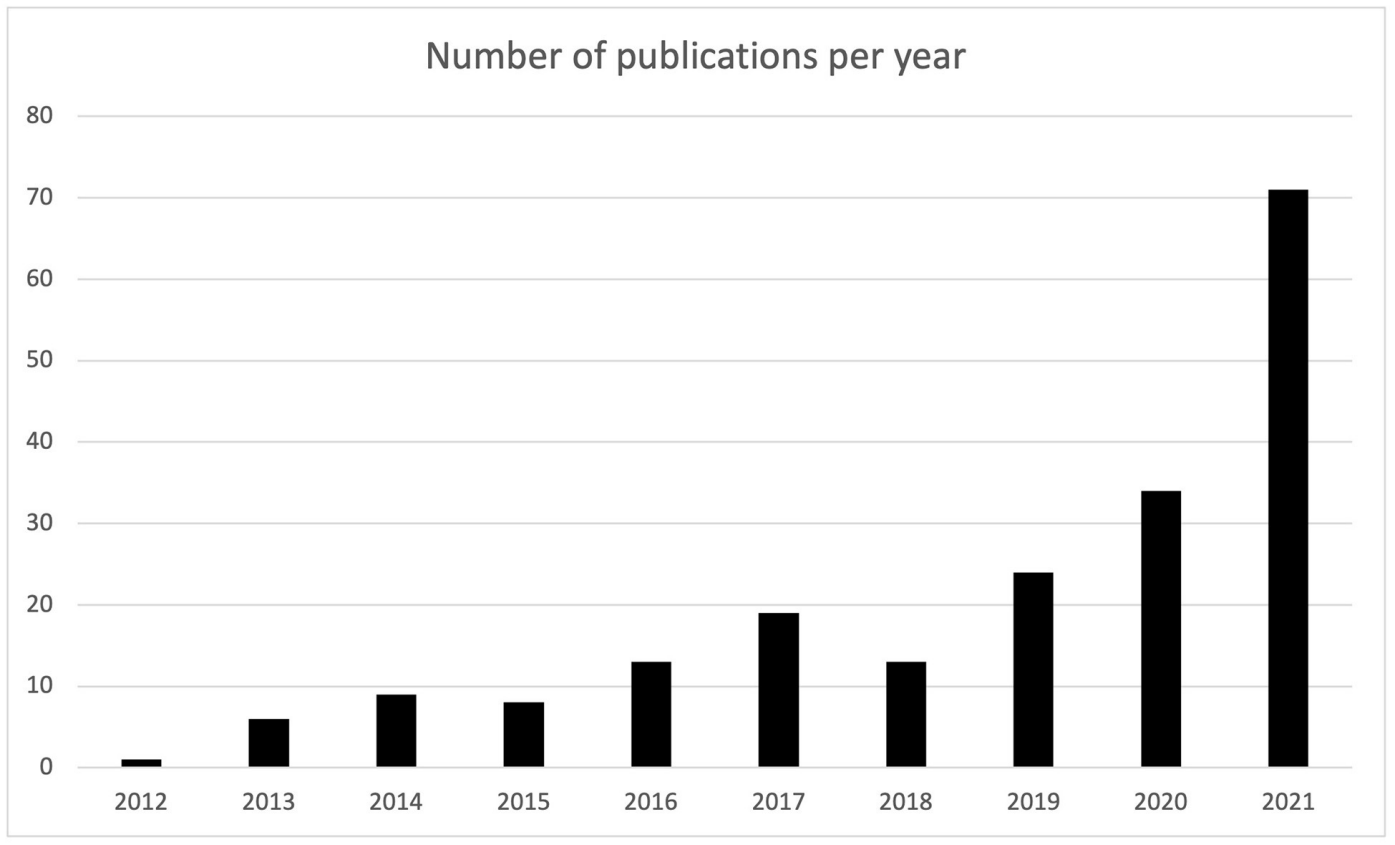
Representation of the number of publication per country.

**Figure 4:**
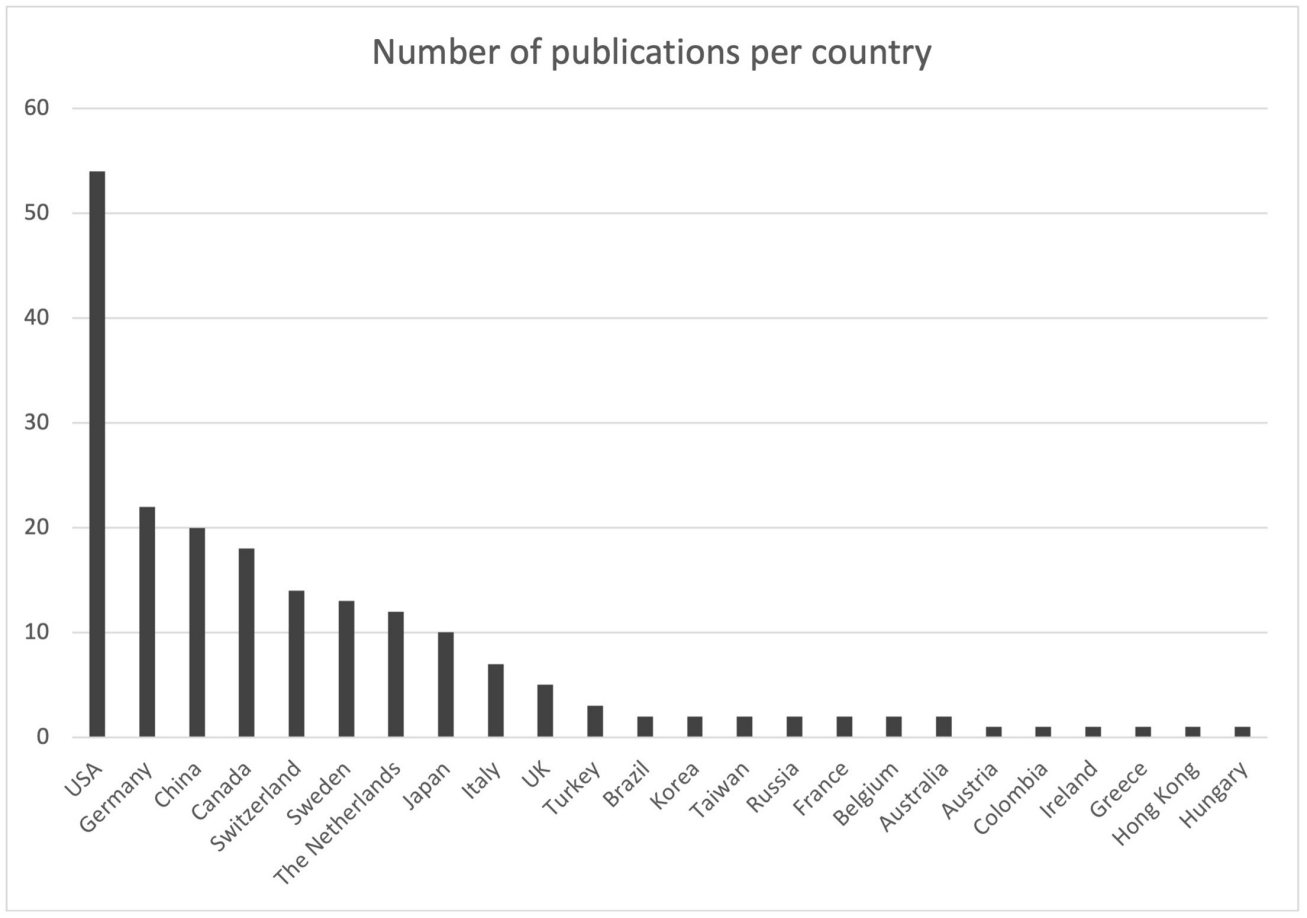
Diagram representing the number of publications per country.

The leading countries, in terms of contribution, were the United States, accounting for 27.3% (*n* = 54) of publications, Germany with 11.1% (*n* = 22), China with 10.1% (*n* = 20) and Canada with 9.1% (*n* = 18) (Figure 3). The contribution from the different countries is presented in Figure 4 and Table 2.

**Table 2:** Number of publications that could be retrieved from the search as grouped by the country of publication.

**Country**	**Number of publications**
USA	54
Germany	22
China	20
Canada	18
Switzerland	14
Sweden	13
The Netherlands	12
Japan	10
Italy	7
UK	5
Turkey	3
Brazil	2
Korea	2
Taiwan	2
Russia	2
France	2
Belgium	2
Australia	2
Austria	1
Colombia	1
Ireland	1
Greece	1
Hong Kong	1
Hungary	1
Grand total	198

### Augmented Reality and Topic

**Figure 5:**
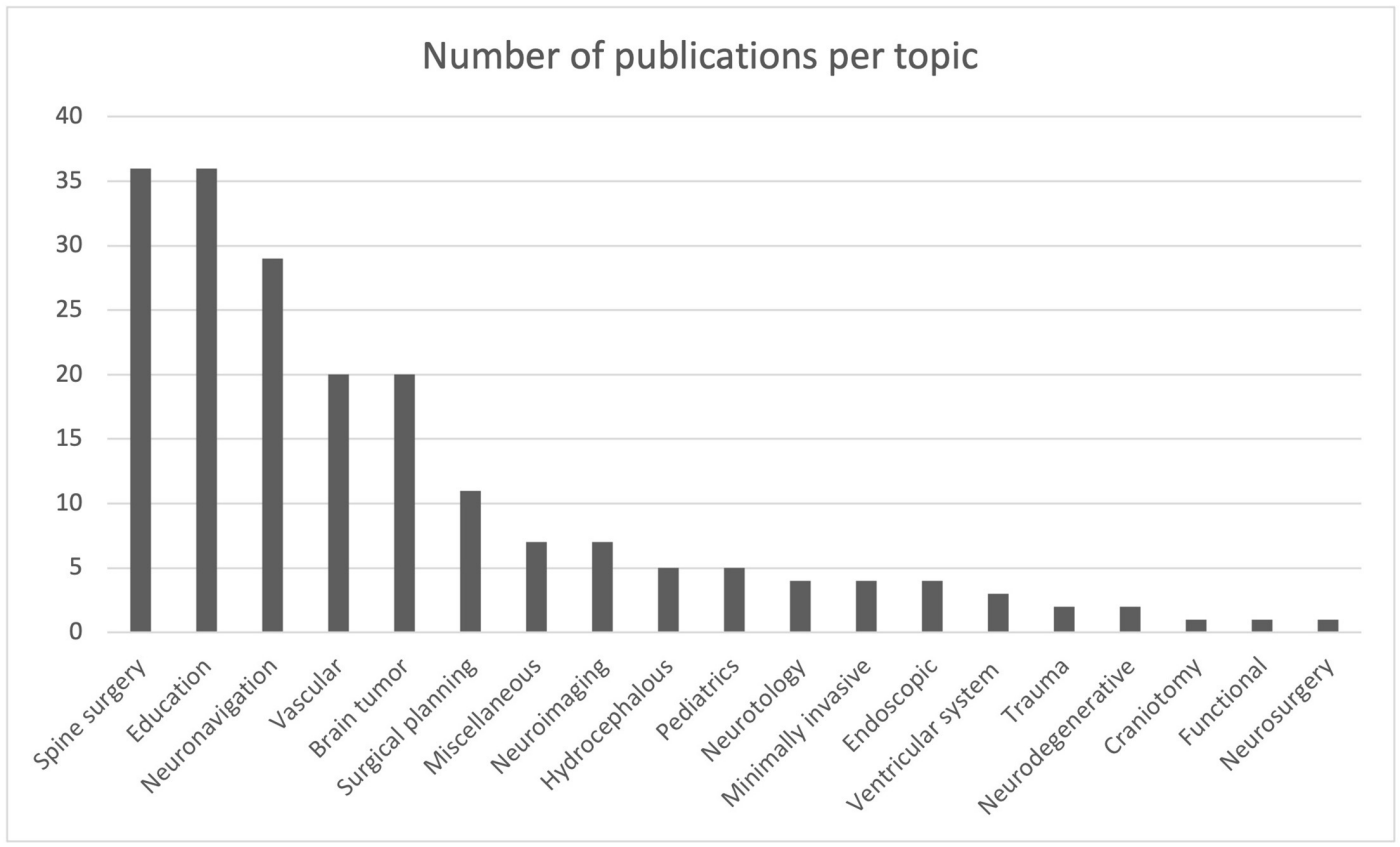
Diagram representing the number of publications per topic.

**Figure 6:**
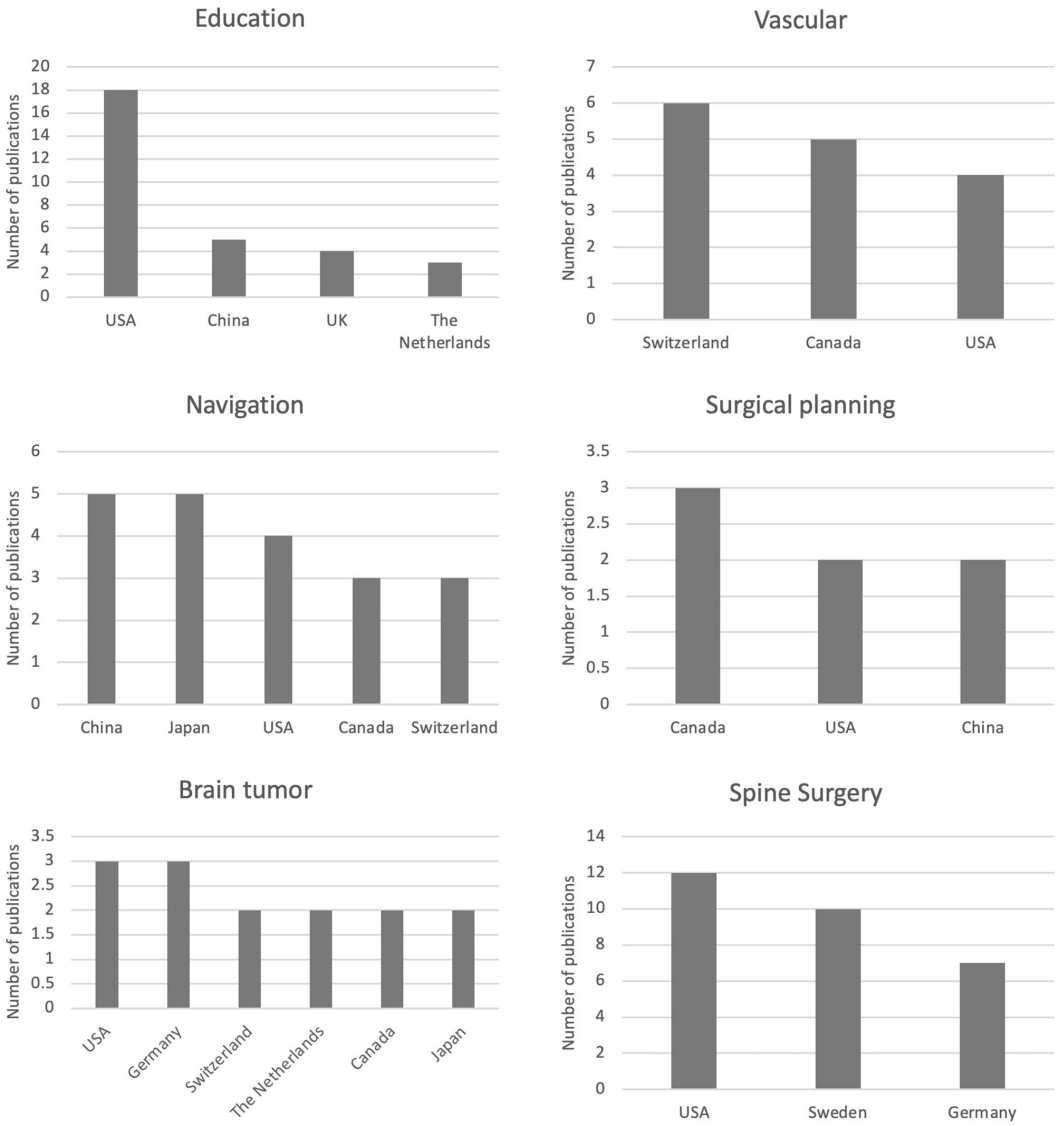
Diagram showing the number of publications per topic per country.

Of the 198 studies, 19 different topics have been detected. The most discussed topics and objects of our analysis are: education, with 36 articles (18.2%), spine surgery, with 36 articles (18.2%), neuronavigation, with 29 articles (14.6%), vascular, with 20 articles (10.1%), brain tumors, with 20 articles (10.1%), and surgical planning, with 11 articles (5.55%) (Figure 5). A correlation was made with each of the included articles (*n* = 198) on the basis of the country of publication and the specific topic (Figure 6). There are a total of 19 different topics. Germany and the USA published articles on 11 different topics, the largest variation among the other countries. The USA published 18 articles concerning education. Spine surgery is the most common topic of publication in Germany (*n* = 7) (Table 3). China focused on 9 different topics, with 5 of those being related to education and another 5 being related to neuronavigation. Canada reported 8 different topics, of which vascular surgery was the most common topic, with 5 articles, followed by surgical planning and neuronavigation with 3 articles.

**Table 3:** Number of publications that could be retrieved from the search as grouped by the field of application.

**Topic**	**Number of publications**
Spine surgery	36
Education	36
Neuronavigation	29
Vascular	20
Brain tumor	20
Surgical planning	11
Miscellaneous	7
Neuroimaging	7
Hydrocephalous	5
Pediatrics	5
Neurotology	4
Minimally invasive	4
Endoscopic	4
Ventricular system	3
Trauma	2
Neurodegenerative	2
Craniotomy	1
Functional	1
Neurosurgery	1
Grand total	198

Thirty-six articles out of the total were related to the education model. The United States published 18 books, followed by China (5 books), the United Kingdom (4), the Netherlands (3), Canada (2), Australia (1), Germany (1), Hungary (1), and Turkey (1). Twenty nine of the total articles (*n* = 198) that were related to spine surgery were published by the USA (*n* = 12), Sweden (*n* = 10), and Germany (*n* = 7). China (*n* = 5) published five articles related to neuronavigation, Japan (*n* = 5), the United States (*n* = 4), Canada (*n* = 3), and Switzerland (*n* = 3) (Table 4).

**Table 4:** Number of publications that could be retrieved from the search as stratified by the country of publication and field of application.

**Country**	**Brain tumor**	**Craniotomy**	**Education**	**Endoscopic**	**Functional**	**Hydrocephalous**	**Minimally Invasive**	**Miscellaneous**	**Neurodegenerative**	**Neuroimaging**	**Neuronavigation**	**Neurosurgery**	**Neurotology**	**Pediatrics**	**Spine surgery**	**Surgical planning**	**Trauma**	**Vascular**	**Ventricular system**	**Grand total**
Australia			1			1														2
Austria											1									1
Belgium						2														2
Brazil																1				1
Brazil																1				1
Canada	2		2							1	3			1	1	3		5		18
China			5	2			1			1	5		1			2	1	1		20
Colombia																1				1
France							1						1							2
Germany	3		1		1				1	2	2			1	7	1		2	1	22
Greece										1										1
Hong Kong	1																			1
Hungary			1																	1
Ireland															1					1
Italy	1	1						1			1				1			2		7
Japan	2								1		5			1	1					10
Korea	1									1										2
Russia	1																1			2
Sweden						1	1				1				10					13
Switzerland	2						1				3				1			6	1	14
Taiwan	2										1									2
The Netherlands	2		3	1				1		1	2				1				1	12
Turkey			1								1				1					3
UK			4	1																5
USA	3		18			1		5			4	1	2	2	12	2		4		54
Grand total	20	1	36	4	1	5	4	7	2	7	29	1	4	5	36	11	2	20	3	198

Eleven articles were published that were related to surgical planning in Canada (*n* = 3), the USA (*n* = 2), and China (*n* = 2). Twenty articles related to vascular were published by Switzerland (*n* = 6), Canada (*n* = 5), and the USA (*n* = 4). Twenty articles that were related to brain tumors were published by the USA (*n* = 3), Germany (*n* = 3), Switzerland (*n* = 2), The Netherlands (*n* = 2), Canada (*n* = 2), and Japan (*n* = 2). The correlation between the number of articles per topic is presented in Figure 6.

### Trends in Neurosurgery

**Figure 7:**
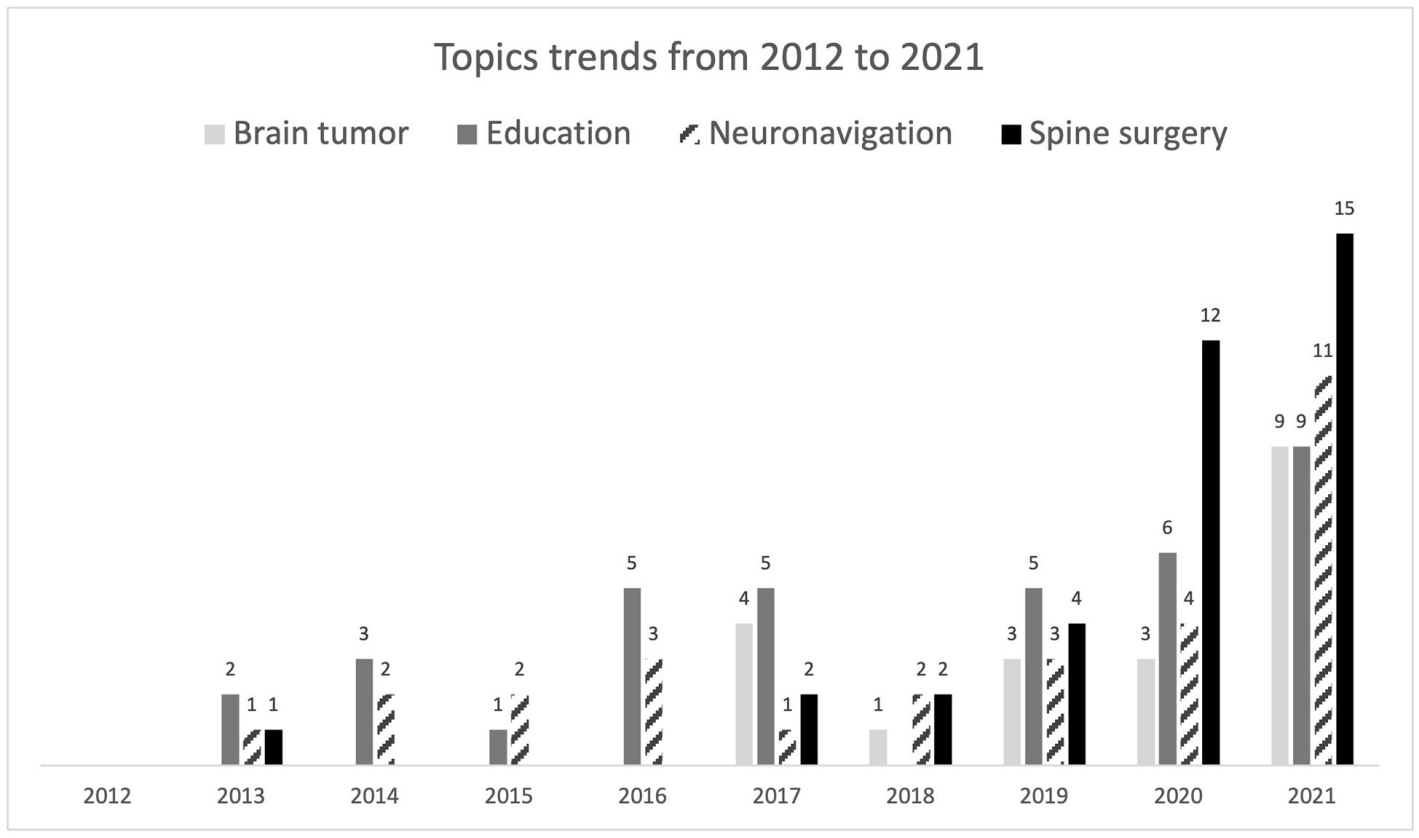
Diagram representing the topics trends per year.

We also investigated the most meaningful trends in neurosurgery applications of AR during the years 2011–2021 (Figure 7). The most remarkable growth was accounted for by spine surgery, which was the most popular topic in 2021 publications, accounting for a total of 15 papers. Spine surgery routinely performs augmented reality-assisted pedicle screw insertion, which is probably the most common intervention nowadays that implements this technology. Neuronavigation is also a growing field in AR-assisted neurosurgery, whose frequency oscillated during the period of study, but it has increased steeply in the last year (2021) with a total of 11 publications (Table 5).

**Table 5:** Number of publications that could be retrieved from the search as stratified by the year of publication and field of application.

**Year**	**Brain tumor**	**Craniotomy**	**Education**	**Endoscopic**	**Functional**	**Hydrocephalous**	**Minimally Invasive**	**Miscellaneous**	**Neurodegenerative**	**Neuroimaging**	**Neuronavigation**	**Neurosurgery**	**Neurotology**	**Pediatrics**	**Spine surgery**	**Surgical planning**	**Trauma**	**Vascular**	**Ventricular system**	**Grand total**
2012																		1		1
2013			2							1	1				1			1		6
2014			3	1							2							3		9
2015			1				1			1	2					1		2		8
2016			5	1		1				1	3					2				13
2017	4		5	1		1	1	1		1	1				2	2				19
2018	1							2		1	2		1	2	2			2		13
2019	3		5				2	1	2	1	3		2		4	1				24
2020	3		6	1							4			1	12	2		5		34
2021	9	1	9		1	3		3		1	11	1	1	2	15	3	2	6	3	69
Grand total	20	1	36	4	1	5	4	7	2	7	29	1	4	5	36	11	2	20	3	198

Surprisingly, despite contributing to a significant portion of publications, AR education research did not increase significantly during the period of study, not even during the first year of the COVID-19 pandemic (2020), when remote learning and alternative technologies were a primary necessity. Only a slight increase was observed in 2021, with a total of 9 publications related to education. We believe it is still not up to the potential of AR. An interesting trend was that of AR-assisted brain tumor surgery, whose number of publications increased in 2021 by more than twice the previous years of study. The non-discussed applications included a small number of articles to make meaningful evaluations, and their trends were generally constant over time, except for vascular surgery, which showed a slight increase in 2020 (5) and 2021 (6). Surprisingly, despite contributing to a significant portion of publications, AR education research did not increase significantly during the period of study, not even during the COVID-19 pandemic (2020–2021), when remote learning and alternative technologies were a primary necessity.

## Discussion

Observation and practice have been the basis of learning in all surgical specialties. Surgery requires a deep knowledge of human anatomy and its variants (10–13). In addition, in surgical practice, it is essential to study the anatomical boundaries, structures, and relationship in three-dimensional arrangement (14–16). For this reason, cadaveric models and expert teaching have been considered the gold standard of medical and surgical education. However, in the last 10 years, surgical simulations and augmented reality tools have appeared on the market and have been implemented in order to try to shorten the learning curve and increase the exposure of trainees to practical training (3). AR is currently being used and tested in a variety of medical specialties and settings throughout the healthcare system. Neurosurgery was among the first medical specialties to implement augmented reality technology into practice (3). For what concerns clinical applications, some fields of neurosurgery are particularly relevant. In fact, imaging modalities such as computed tomography and magnetic resonance are indeed crucial in current practice for optimal pre-operative planning in order to determine the optimal approach for the surgery, especially in complex skull base approaches (17–23). AR is used for guidance of screw insertion in pedicles during minimally invasive spinal surgery. Additionally, AR finds application in vascular neurosurgery: some software has been developed for the treatment of cerebral aneurysms and also in the endovascular field for the correct selection of intracranial stents in the most complex conditions or for the practice of young interventionalists (24–27).

In view of the increasingly widespread applicability of AR in neurosurgery, the results of our analysis are not surprising. The number of publications concerning AR has increased steadily in the last 10 years, especially in the last 3 years. This reflects the fact that technologies are more and more included and upgraded in surgical and clinical practice nowadays. The technologies at the base of AR are more easily shared among various neurosurgeons, allowing easy diffusion of the use of these techniques and a consequent increase in scientific production.

The geographic distribution of the implementation of augmented reality technologies in neurosurgery shows a prominence in the USA, Canada, China, and Germany. The USA not only has the highest number of publications on augmented reality but is also characterized by a greater selection of topics than Germany. Furthermore, a constant geographical distribution is observed among the various topics, with the exception of augmented reality in the field of education. The USA has the most extensive adaptation of augmented reality in the training of medical students, residents, and surgeons. Asia deserves a special mention for the use of AR for intraoperative navigation. Despite only 12.6% of the total publication comes from Asian countries, especially China, it should be stated that the large majority of them has been published in the most recent years, showing an important increase in interest that will likely to continue in the next years.

Spine surgery, with the increased use of minimally invasive techniques and the use of intraoperative neuronavigation, has led to a significant increase in the application of AR, especially in the USA, Germany, and Sweden. Finally, Canada offers a major contribution in AR applied to the surgical plans.

AR offers a magnitude of potential advantages to the training of neurosurgeons as it sets up good short processing times and allows training and practice of major neurosurgical procedures outside the operation room (21–23). AR offers a training method with a practical framework by providing a protected training environment (28). This is important as it integrates training and further development of the surgery curriculum that will ultimately lead to a significant reduction in the cost of training.

Current standard training processes will outline areas of further development and improvement by augmented reality technologies. Furthermore, AR technologies represent an innovative learning medium that enables trainees to have a flexible, on- demand training directive that will enhance their curriculum (3, 8, 28). Different teaching and learning objectives can be achieved through the use of this technology in training, with a special focus on practical learning scenarios in a medical or surgical environment. Direct measures to optimize education and training are needed to meet the requirements of digitalization in neurosurgery and be more successful (22). Conversely, it is recommended that the adapting department have a transparent goal before considering the implementation of augmented reality in training. Moreover, when implementing augmented reality devices in training, the objective and the expectations of the application in training must first be defined. This is imperative because not every augmented reality device can meet the requirements of the planned neurosurgical training curriculum. Furthermore, the requirements in a surgical setting are based on robustness and usability, which enhances technological possibilities such as 3D imaging in a clinical scenario. Therefore, recommending the technology to training departments to determine areas in which specific processes need to be achieved by the device is imperative. Lastly, the framework of implementing such devices in the training and work of surgeons needs to be stratified on an outgoing successful outcome basis for the predetermined goals of training (28).

Furthermore, the adaptation of AR applications in neurosurgery benefits training and simulation due to the creation of a no-risk virtual environment where surgeons can develop and refine skills through harmless repetition since neurosurgery carries a very small margin of error during surgery (21, 22, 29). Conversely, the use of AR in neurosurgery carries some limitations. Primarily, AR applications may result in a delay in the display or projection of the images in AR neuronavigation. This is problematic as it provides inaccurate localization to the user. Secondarily, a major challenge in the use of AR applications is image alignment due to inaccurate calibration and optical distortions that alter the image. It is also important to highlight that tissue movement is a challenge in all AR applications as the movement of tissue during surgery increases the error in image alignment intra-operatively (22, 23), there are also other points to consider. It is likely that AR will have an important role in image-based augmentation of the surgical environment. This will require increasingly powerful microcomputers to drive AR, which is currently limited but will improve with time. For the device to be a natural extension of the surgeon's senses, it has to be light, mobile, comfortable and functional for potentially long periods of time. Therein lies the limitations of the technology at present, where the battery life is limited, devices are large and the cables can be cumbersome. Such technology has to progress at present and eventually after several generations of development these tools will become as common as surgical loupes (30).

Although augmented reality promises to become an essential part of the future of neurosurgical practice, major challenges have yet to be solved. The two most serious have been registration errors and system delays, which have hampered the use of AR in the most delicate procedures, such as skull base surgery and any other operation requiring sub-millimetric precision There has been a great effort from the scientific community in trying to address each of these challenges, and in many cases, very promising solutions have been proposed. However, even though it is true that these problems will be partially solved by the ever-evolving progress of technologies, from our investigation of the reviewed articles, we hypothesized that greater synchronization among the most active centers can significantly accelerate this process. Most of the current AR hardware is custom-made and difficult to distribute (31). This not only renders the technology hardly accessible, but it also implies that the research and strategies to solve the above-mentioned challenges are specifically oriented to the customized device that is being developed by the laboratory of interest. Such a lack of synchronization among institutions has limited the impact of individual findings and occasionally led to some confusion. It was, however, possible to find a promising solution for each of the mentioned challenges by investigating more deeply. This means that a more dynamic collaboration among the countries can truly benefit from these advancements, as was also suggested by some authors (32). Furthermore, enhancing the agreement among different centers can also help clarify the evaluation criteria of these technologies. One of the major pillars of our study was not only to investigate how AR will advance neurosurgical care in the developed world, but also how it could impact the underdeveloped world, where these technologies are unimaginable. No publications could indeed be retrieved from Africa and other developing areas. Addressing how AR could improve the local healthcare system in these countries is an extremely delicate topic, since many such communities are not yet ready to sustain the complementary technologies that go along with AR implementations. However, AR could be a superb tool that the developed world can offer to underdeveloped areas to accelerate and refine the learning and training of simple and large-scale lifesaving procedures, even in the limited time duration of global neurosurgery missions. AR has been repeatedly shown to reduce the learning curves and bridge the expertise gap between students and senior neurosurgeons (33–35). AR could similarly make a difference, even in developing countries.

### Study Limitations

Despite the authors' best efforts, the present study exhibits some limitations. Publication limitations may have been present due to the inclusion of studies published only in English. In addition, the included studies were extremely heterogeneous by including multiple augmented reality systems.

## Conclusion

Augmented reality is still mostly used for education, surgical planning, and neuronavigation. This technology has also been implemented in clinical practice; in the last 10 years, we have observed an exponential increase in the application of augmented reality, especially in spinal surgery. Given the continuous advancement of augmented reality techniques and their increasing popularity, it may be possible to develop a unified education plan for future neurosurgeons. Countries with limited facilities could possibly benefit only if it's coupled with a specific target in this education model.

## Data Availability Statement

The original contributions presented in the study are included in the article/supplementary material, further inquiries can be directed to the corresponding author/s.

## Author Contributions

DC, IZ, and FS: concept and design. AS, AJ, ACo, ABi, KS, ACB, and AP: acquisition of data. DC, AS, AJ, ACo, AB, KS, ACB, and AP: analysis and interpretation of data. All authors: drafting the article and critically revising the article. All authors contributed to the article and approved the submitted version.

## Conflict of Interest

The authors declare that the research was conducted in the absence of any commercial or financial relationships that could be construed as a potential conflict of interest.

## Publisher's Note

All claims expressed in this article are solely those of the authors and do not necessarily represent those of their affiliated organizations, or those of the publisher, the editors and the reviewers. Any product that may be evaluated in this article, or claim that may be made by its manufacturer, is not guaranteed or endorsed by the publisher.
